# Application of Handheld Tele-ECG for Health Care Delivery in Rural India

**DOI:** 10.1155/2014/981806

**Published:** 2014-09-28

**Authors:** Meenu Singh, Amit Agarwal, Vineet Sinha, Rohit Manoj Kumar, Nishant Jaiswal, Ishita Jindal, Pankaj Pant, Munish Kumar

**Affiliations:** ^1^Advanced Pediatric Centre, Postgraduate Institute of Medical Education and Research, Sector 12, Chandigarh 160012, India; ^2^ICMR Centre for Evidence Based Child Health, Advanced Pediatric Centre, Postgraduate Institute of Medical Education and Research, Sector 12, Chandigarh 160012, India; ^3^Department of Electronic Division, Bhabha Atomic Research Center, Mumbai 400085, India; ^4^Department of Cardiology, Postgraduate Institute of Medical Education and Research, Chandigarh 160012, India

## Abstract

Telemonitoring is a medical practice that involves remotely monitoring patients who are not at the same location as the health care provider. The purpose of our study was to use handheld tele-electrocardiogram (ECG) developed by Bhabha Atomic Research Center (BARC) to identify heart conditions in the rural underserved population where the doctor-patient ratio is low and access to health care is difficult. The objective of our study was clinical validation of handheld tele-ECG as a screening tool for evaluation of cardiac diseases in the rural population. ECG was obtained in 450 individuals (mean age 31.49 ± 20.058) residing in the periphery of Chandigarh, India, from April 2011 to March 2013, using the handheld tele-ECG machine. The data were then transmitted to physicians in Postgraduate Institute of Medical Education and Research (PGIMER), Chandigarh, for their expert opinion. ECG was interpreted as normal in 70% individuals. Left ventricular hypertrophy (9.3%) was the commonest abnormality followed closely by old myocardial infarction (5.3%). Patient satisfaction was reported to be ~95%. Thus, it can be safely concluded that tele-ECG is a portable, cost-effective, and convenient tool for diagnosis and monitoring of heart diseases and thus improves quality and accessibility, especially in rural areas.

## 1. Introduction

Cardiovascular disease (CVD) is estimated to be the commonest cause of death as well as disability in India by 2020 as stated in the World Health Report 2002 [1]. According to the Global Status Report on Noncommunicable Diseases (2011) published by the World Health Organization, CVD resulted in more than 2.5 million deaths in India in 2008 [2]. The prevalence of coronary heart disease, from multiple epidemiological studies, is estimated to be between 7 and 13% in urban and between 2 and 7% in rural India [3]. Recently, a reversal in trend has been observed with rural areas accounting for an equal, and sometimes higher, burden of CVD and its risk factors. This is analogous to the manifestation of socioeconomic gradient noted in developed countries [4–6].

India faces the challenge of ensuring health care, especially in the rural areas, plagued with resource shortage and nonavailability of doctors. Early diagnosis and treatment are crucial to ensure sustainable medical treatment and improved survival rates [7]. Telemedicine serves as a promising cost-effective alternative in light of the fact that an early, tailored intervention has been shown to prevent deaths and improve functional recovery [8].

Telemedicine is defined by the American Telemedicine Association as “the use of medical information exchanged from one site to another via electronic communications for health and education of the patient or healthcare provider and for the purpose of patient care” [9]. Telemonitoring enables transmission of data, pertaining to diagnostic investigations, to a remote site for medical consultation [8, 10–12]. There is considerable evidence that the utility of telemonitoring as a diagnostic tool is equivalent to that of traditional hospital examination [13–17].

Tele-ECG is a convenient tool to distinguish individuals with suspected heart diseases that may require urgent referral to a hospital or even emergency medical services [18]. It utilizes m-Health technology, which involves the use of mobile phones for data transmission, so as to provide health care services to remote areas [19].

The aim of the study was to report the design and implementation strategy of handheld tele-ECG as well as its use in the periphery and impact of telemedicine in the delivery of health care services.

## 2. Methodology

### 2.1. Subjects

Four hundred and fifty individuals (10% dropout rate) from different villages in the outskirts of Chandigarh were enrolled for this study during the period from April 2011 to March 2013 (Table 1). A complete history and physical examination were performed and written informed consent was obtained. The Ethics Committee of Postgraduate Institute of Medical Education and Research approved this study (PGI/IEC/2011/725-26).

### 2.2. Methods

The detailed history and clinical examination were entered into an electronic patient record.* Inclusion criteria*. The individuals were included who (patient/caretaker) were agreed for the home visits by the monitoring team, suitable social circumstances for home care, appropriate degree of home support if living alone and reside in the project catchment area.

*Exclusion Criteria.* The individuals were excluded if they had hemoptysis (spitting up blood from the respiratory tract), pneumothorax (free air or gas in the pleural cavity), history of recent heart attack, unstable angina, aneurysm (cranial, thoracic, or abdominal), thrombotic condition (such as clotting within a blood vessel), and recent thoracic or abdominal surgery and those who refused to give consent.

### 2.3. Procedure of Tele-ECG

**Figure 1:**
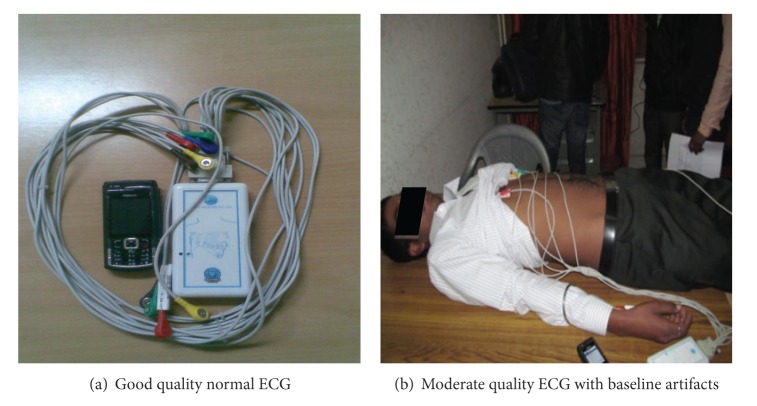
Field testing by ECG.

The ECG is a noninvasive test that is used to reflect underlying heart conditions by measuring the electrical activity of the heart. A handheld tele-ECG instrument developed by BARC, operated with the help of a mobile phone via Bluetooth, was used for performing tele-ECG (Figure 1). ECG leads were attached to the body while the patient lied flat on a bed. These leads were attached to each extremity (four in total) and to six predefined positions in front of the chest. A small amount of gel was applied to the skin, which allowed the electrical impulses of the heart to be more easily transmitted to the ECG leads. The leads were attached by electrodes or by small adhesive patches attached loosely to the skin. The test took about five minutes and was painless.

The handheld tele-ECG has a unique feature of recording ECG of the subject and displaying the same on the mobile screen. After complete recording, the ECG could be sent to an expert's mobile through multimedia messaging service (MMS) for his opinion. It was performed in a rural setting in the periphery of Chandigarh. The handheld tele-ECG is a low cost, portable, and compact screening tool. It also has mobile as well as LAN connectivity. It provides acquisition, processing, storing, and visualization of ECG in real time by using a secure GPRS connection for transfer of ECG data.

## 3. Results

Four hundred and fifty individuals with mean age in years (31.49 ± 20.05) in the community setting were recruited for the study (Table 2). Handheld tele-ECG was used to obtain an ECG from these individuals. This technology has been developed at the electronic division of BARC and has been validated in a pilot project at PGIMER, Chandigarh, in 2009. In the above-mentioned pilot study, 50 individuals were enrolled who underwent both tele-ECG and conventional ECG. Interpretation of results of ECG was similar by both methods with 99% correlation. Our aim, in the present study, was to validate the handheld tele-ECG in the community health setting as a screening tool for a plethora of cardiac conditions.

**Figure 2:**
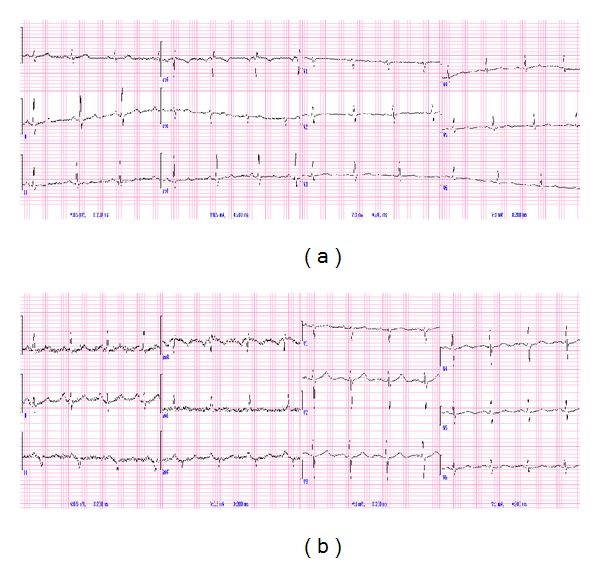
Quality of transmitted ECG.

We observed transmission rate from the tele-ECG to mobile phone was 100%, although the quality was graded to be either moderate or good (Figure 2). Moderate quality transmission due to noise or baseline artifacts was noted in twenty percent of individuals (90/450) while the rest showed good quality transmission (360/450).

**Figure 3:**
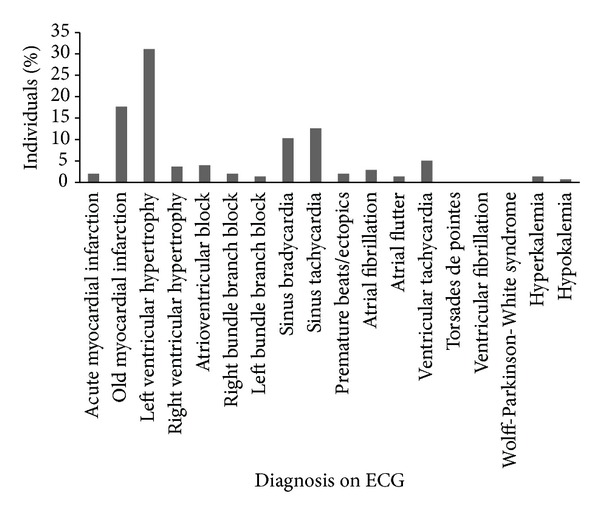
Distribution of abnormalities detected on tele-ECG.

An expert at the tertiary care centre, PGIMER, read the transmitted electrocardiograms from the remote centres. The rate, rhythm, axis, intervals, P wave, QRS complex, ST segment, and T wave changes, if any, were noted. ECG was reported as normal in 70% individuals. A myriad of abnormalities were noted in the remaining 30% individuals (Figure 3, Table 3). Of these, the majority was left ventricular hypertrophy (9.3%) followed by old myocardial infarction (5.3%). Sinus bradycardia and sinus tachycardia were reported in a similar proportion of individuals (3.1% and 3.7%, resp.). ECG findings matched with the history of the patient except three individuals who showed acute myocardial infraction. Interpretation of ECG did not reveal any individuals with abnormalities, namely, ventricular fibrillation, Torsades de pointes, or Wolff-Parkinson-White syndrome.

Individuals with abnormal results were referred to PGIMER. Further monitoring of the same individuals with a standard ECG machine was performed. Outcomes were noted to be similar for both handheld tele-ECG and standard ECG which is outlining the reliability and accuracy of handheld tele-ECG. Individualized treatment was instituted for each patient accordingly. Patients reported ~95% satisfaction with the system.

## 4. Discussion

Telemonitoring enhances the health care delivery in underserved communities by facilitating access to diagnostic tests as well as increasing communication between primary care practitioners and specialists in tertiary care centres [20, 21]. Tele-ECG is a convenient, sustainable, and reliable means of monitoring cardiac function. It ensures timely physician-patient contact in emergency situations while avoiding unnecessary hospital visits [22–27]. Hence, the field of emergency medicine can be revolutionized by scientific evaluation and implementation of telemedicine systems [28].

The present study demonstrates the applicability of a remote care model that ensures improved availability of health care resources to populations residing in distant areas. The handheld tele-ECG is an indigenous design that shows immense potential in replacing conventional ECG, especially for screening purposes in the remote areas. This is in light of the fact that the pilot study showed 99% correlation between tele-ECG and conventional ECG. Moreover, a previous studyreported 76% consistently normal ECGs [29], similar to 70% outlined in our study.

Our study had 80% good quality ECG and only 20% were of moderate quality that is comparable to existing literature, both of which had 77% good quality transmission [29, 30] but lesser than Alte et al. who reported 94% good ECGs [31]. The transmission rate in our study is 100%, only reported in one other study that showed no technical difficulties or transmission failures [32]. ECG transmission failure occurred in 14% individuals in the study by Terkelsen et al. [33] whereas the TIME-NE study reported failure in 44% patients [34].

Tele-ECG can bring a revolution to screening large populations for a variety of cardiac conditions and can be potentially lifesaving. Early detection of acute myocardial infarction by tele-ECG and transmission of information to the attending emergency physician can accelerate management of the patient [35, 36]. Patients with preexisting coronary artery disease and chronic heart failure can be managed safely and effectively with concomitant telemedicine, as it shows potential to enhance quality of life and improve prognosis [10]. These services have shown to meet the patient's expectations as judged by high acceptance and patient satisfaction [29]. This is in concordance with our study, which also reports ~95% patient satisfaction. This model can also be used for self-monitoring of individuals suffering from heart conditions residing in urban areas. This will lead to decreased hospital patient load and improved services to needy patients. Furthermore, a home-based cardiac consultation for chronic heart disease patients might even be possible in the future.

Even though the above-mentioned model has several advantages, certain hurdles still need to be overcome. Physician acceptance is low due to the shift from in-house to telephonic consultations [29]. Protection of data privacy is another critical issue that needs to be addressed. A study linked ECG to a person only by two link tables, thus ensuring data privacy [31]. There is a possibility of erroneous diagnosis as well as subjective differences in interpretation of tele-ECG by different medical readers. Thus, it is preferable to obtain a computer-based analysis, for example, using the MEANS algorithm for the 12-lead ECG [37, 38], but even that is associated with false negative results, requiring additional reading by medical personnel [39].

Thus, our study concludes that tele-ECG is an excellent model to curb morbidity and mortality resulting from cardiovascular diseases. Epidemiologically, it can have a significant impact on the policy decisions of a nation [40, 41], as it has a vast untapped potential of improving the quality of health care in rural areas, increasing access to specialists in referral centres, reducing transportation of patients to doctors, supporting primary care physicians, and overcoming shortage of doctors, especially in remote areas.

Further use of this technology can be utilized to build similar devices, for epidemiological diseases of epic proportions, which can provide support and build infrastructure in areas where resources are scarce. Transmission of the data gathered from such devices to referral centres for an expert opinion is an effective, economically viable, and technically feasible model of health care delivery in far-flung areas. A previous study compared the transmission of a 12-lead ECG which recorded and transmitted to a cardiac centre versus standard ECG recorded at the same time from the same patient. They found the quality of tele-ECG was adequate to diagnose 98% patients [18]. Further enhancement of this prototype to develop autotransmission of data to predestined centres can initiate the process of activating health care professionals in tertiary centres to screen high risk population and guide further management plan. These systems can pave the way for a new era of medicine where health care delivery to each and every individual, even in remote areas, is a realistic possibility.

## Figures and Tables

**Table 1:** Enrolment of subjects at different centres (Chandigarh, India).

S. No.	Name of the centre	Number of subjects enrolled
1	Khuda Ali Sher	50
2	Sarangpur	60
3	Dhanas	54
4	Pulsar Gurudwara	87
5	Kaimbwala	21
6	Navjeevan Church	178

**Table 2:** Individual characteristics.

S. No.	Patient characteristics	
1	Age (years) (mean ± SD)	31.49 ± 20.05
2	Height (cm) (mean ± SD)	157.28 ± 16.16
3	Weight (Kg) (mean ± SD)	54.35 ± 17.06
4	Sex M/F	212/238

**Table 3:** Interpretation of handheld tele-ECG.

S. No.	Diagnosis on ECG	*N* (%)
1	Normal	315 (70)
2	Acute myocardial infraction	3 (0.6)
3	Old myocardial infarction	24 (5.3)
4	Left ventricular hypertrophy	42 (9.3)
5	Right ventricular hypertrophy	5 (1.1)
6	Atrioventricular block	6 (1.1)
7	Right bundle branch block	3 (0.6)
8	Left bundle branch block	2 (0.4)
9	Sinus bradycardia	14 (3.1)
10	Sinus tachycardia	17 (3.7)
11	Premature beats/ectopic	3 (0.6)
12	Atrial fibrillation	4 (0.8)
13	Atrial flutter	2 (0.4)
14	Ventricular tachycardia	7 (1.5)
15	Torsades de pointes	0 (0)
16	Ventricular fibrillation	0 (0)
17	Wolff-Parkinson-White syndrome	0 (0)
18	Hyperkalemia	2 (0.4)
19	Hypokalemia	1 (0.2)
